# Endoscopic Sphincterotomy before Fully Covered Metal Stent Placement Is Not Required for Distal Malignant Biliary Stricture due to a Pancreatic Head Tumor

**DOI:** 10.1155/2019/9675347

**Published:** 2019-01-15

**Authors:** Kazunari Nakahara, Yosuke Michikawa, Ryo Morita, Keigo Suetani, Nozomi Morita, Junya Sato, Kensuke Tsuji, Hiroki Ikeda, Kotaro Matsunaga, Tsunamasa Watanabe, Nobuyuki Matsumoto, Shinjiro Kobayashi, Takehito Otsubo, Fumio Itoh

**Affiliations:** ^1^Department of Gastroenterology and Hepatology, St. Marianna University School of Medicine, 2-16-1 Sugao, Miyamae-ku, Kawasaki 216-8511, Japan; ^2^Department of Gastroenterological and General Surgery, St. Marianna University School of Medicine, 2-16-1 Sugao, Miyamae-ku, Kawasaki 216-8511, Japan

## Abstract

**Background/Aims:**

Endoscopic sphincterotomy (EST) is often performed before fully covered self-expandable metal stent (FCSEMS) placement in order to prevent pancreatitis. However, it is not clear whether EST prevents pancreatitis or affects other adverse events (AEs). This study is conducted to evaluate the necessity of EST before FCSEMS placement for distal malignant biliary strictures due to a pancreatic head tumor.

**Methods:**

This study included 68 patients who underwent FCSEMS placement for distal malignant biliary stricture due to a pancreatic head tumor. Treatment outcomes and AEs were retrospectively compared between 32 patients with EST before FCSEMS placement (EST group) and 36 patients without EST (non-EST group).

**Results:**

The success rates of drainage for the EST and non-EST groups were 100% and 97.2%, respectively (*P* = 0.95). The incidence of pancreatitis in the EST and non-EST groups was 3.1% and 0%, respectively (*P* = 0.95). The incidence of hyperamylasemia in the EST and non-EST groups was 12.5% and 13.9%, respectively (*P* = 0.85). The incidence of all AEs in the EST and non-EST groups was 15.6% (pancreatitis: 1, cholecystitis: 2, and stent migration: 2) and 13.9% (cholecystitis: 3, stent migration: 2), respectively (*P* = 0.89).

**Conclusions:**

EST before FCSEMS placement for distal malignant biliary stricture due to a pancreatic head tumor does not affect the successful drainage and incidence of adverse events. The necessity of EST to prevent pancreatitis before FCSEMS placement was deemed low.

## 1. Introduction

Covered self-expandable metal stents (CSEMS) potentially have a longer patency and have been widely used for unresectable malignant distal biliary strictures [1–5]. Fully covered self-expandable metal stents (FCSEMS), a type of CSEMS that is covered over its entire length, are useful in terms of reintervention for their removability in the case of stent occlusion [6–10]. However, the risks associated with CSEMS placement emerge as a concern: the orifice of the pancreatic duct can possibly become clogged to cause pancreatitis due to the outflow obstruction of pancreatic juice when the CSEMS is placed across the duodenal papilla [11]. Therefore, endoscopic sphincterotomy (EST) is often performed before CSEMS placement in order to prevent pancreatitis from preserved pancreatic juice outflow by separating the pancreatic duct and bile duct orifices [12–14]. However, some studies have reported that EST before CSEMS placement does not contribute to the prevention of pancreatitis [15–22]. Two prospective randomized controlled trials (RCTs) both showed a lack of efficacy of EST in the prevention of pancreatitis: the incidence of pancreatitis was compared in the presence and absence of EST before CSEMS placement [18, 19]. The CSEMS used in these studies were, however, partially covered self-expandable metal stents (PCSEMS), while FCSEMS was not used. The use of FCSEMS, which is fully covered along its entire length, is considered to be associated with an increased risk of pancreatitis due to occlusion of the pancreatic duct orifice as compared with PCSEMS, which is uncovered along its distal sides. The safety of placing FCSEMS without performing EST prior to the procedure, however, has not been clarified.

Therefore, although RCTs on the necessity of EST before placement of PCSEMS have already been reported [18, 19], we conducted the following confirming studies focused on FCSEMS. We report here the first study focused on FCSEMS that assesses the necessity of EST before stent placement in cases with a distal malignant biliary stricture due to a pancreatic head tumor.

## 2. Materials and Methods

### 2.1. Patients

We selected 68 patients having a distal malignant biliary stricture due to a pancreatic head tumor who underwent a transpapillary placement of FCSEMS under endoscopic retrograde cholangiopancreatography (ERCP) at the St. Marianna University School of Medicine between January 2010 and December 2017. There were 33 patients who underwent FCSEMS placement as an initial drainage. In 35 patients, previous biliary drainage had been performed before FCSEMS placement (endoscopic biliary drainage: 25, endoscopic nasobiliary drainage: 9, and percutaneous transhepatic biliary drainage: 1). We excluded patients having a pancreatic head tumor associated with a lack of dilatation of the main pancreatic duct (MPD) and patients who received a postoperative reconstruction other than a Billroth I reconstruction. An MPD less than 3 mm in diameter on image findings by any of the abdominal computed tomography, magnetic resonance cholangiopancreatography, and ERCP was defined as no MPD dilatation. The total patients included 37 males and 31 females, with the mean age of patients being 73.5 ± 11.5 years (mean ± standard deviation (SD)). Sixty-four patients had pancreatic head cancer and the other four patients had a metastatic pancreatic tumor.

**Figure 1:**
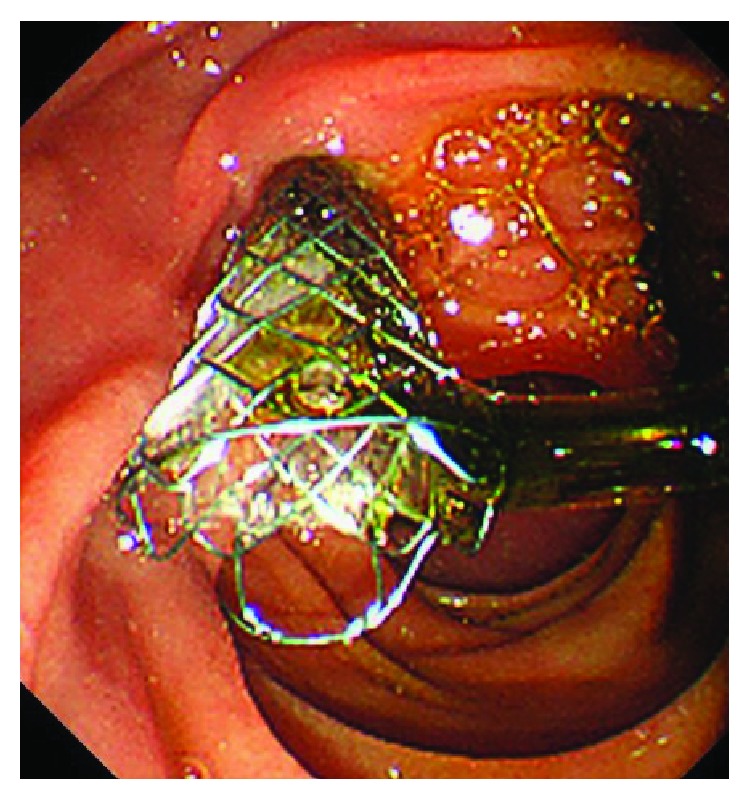
A fully covered self-expandable metal stent was placed across the papilla with approximately 1 cm of the distal end of the stent protruding into the duodenal lumen.

We defined 32 patients who underwent EST before FCSEMS placement as the EST group, and 36 patients who did not as the non-EST group. Each attending endoscopist judged and decided at the time of the procedure whether or not to perform EST, which was basically not performed in patients with a coagulation disorder or who were medicated with oral anticoagulants. In the EST group, EST was performed at the same ERCP session as FCSEMS placement in 14 patients and EST had been performed during the previous ERCP session in 18 patients. EST was performed using high-frequency devices: ICC 200 (Erbe Elektromedizin Corp., Tuebingen, Germany; 120 W, EndoCut mode effect 3) or ESG-100 (Olympus, Japan; 50 W, PulseCut Slow mode). Each FCSEMS was placed across the duodenal papilla with approximately 1 cm of the distal end of the stent protruding into the duodenal lumen in all patients (Figure 1). The FCSEMS used were a WallFlex Biliary RX stent (Boston Scientific, Marlborough, MA, USA) in 51 patients, a Niti-S COMVI stent (Taewoong Medical Inc., Goyang, South Korea) in eight patients, an X-Suit NIR Biliary Metal stent (Olympus Medical Systems Corp., Tokyo, Japan) in three patients, a Hanarostent (M.I. Tech, Seoul, South Korea) in three patients, a ZEO stent (Zeon Medical Inc., Tokyo, Japan) in one patient, a Bonastent (Sewoon Medical Co. Ltd., Seoul, South Korea) in one patient, and a Niti-S SUPREMO-12 stent (Taewoong Medical Inc., Gimpo, South Korea) in one patient. Stent lengths were 6 cm in 46 patients and 8 cm in 21 patients, with stent diameters being 10 mm in 67 patients and 12 mm in one patient. All ERCP procedures were performed under the supervision of experts experienced in more than 2000 ERCP procedures.

In all patients, gabexate mesilate (600 mg/day) or ulinastatin (150000 IU/day) was administered on the day of FCSEMS placement for the prevention of post-ERCP pancreatitis. No patients received nonsteroidal anti-inflammatory drugs suppository for the prevention of post-ERCP pancreatitis in this study. A blood test was undertaken before FCSEMS placement, three hours after the procedure, the next day, and two days later in all patients.

### 2.2. Measurements

We retrospectively compared patients' backgrounds, endoscopic procedures, stent type, success rate of drainage, incidence of pancreatitis, incidence of hyperamylasemia, change in the serum amylase level, and the incidence and details of all adverse events (AEs) between the EST (*n* = 32) and non-EST groups (*n* = 36). In this study, the primary outcome was to evaluate the necessity of EST before FCSEMS placement to prevent post-ERCP pancreatitis and the secondary outcome was to evaluate the clinical benefits and disadvantages of EST before FCSEMS placement.

The length of an EST incision was defined as small (up to the proximal hooding fold), medium (between small and large), or large (up to the superior margin of the sphincter opening). The success of drainage was defined as a decrease in the serum total bilirubin level to 3 mg/dL or lower or having half or less than the previous value within 2 weeks after FCSEMS placement. In the patients where the serum bilirubin levels had already decreased at the time of FCSEMS placement by previous biliary drainage, we defined the absence of increase in serum bilirubin levels after FCSEMS placement as a successful drainage. The serum amylase level (normal range: 37–124 IU/L) was determined by blood tests performed before FCSEMS placement, 3 hours after the procedure, the next day, and two days later. The diagnosis and severity assessment of AEs, including pancreatitis, bleeding, perforation, and cholangitis, were undertaken according to the consensus guidelines proposed by Cotton et al. [23]. Hyperamylasemia was defined as an increase in the serum amylase level to three-fold or higher of the normal limit (>372 IU/L) without associated abdominal pain after FCSEMS placement.

This study was approved by the institutional review board of St. Marianna University School of Medicine (approval number: 3903).

### 2.3. Statistical Analysis

Chi-square test, Fisher's exact test, and Welch's *t* test were used for statistical analysis, where appropriate. A *P* value of <0.05 was regarded as significant. Statistical analysis was performed using StatMate IV software (ATMS Co. Ltd., Tokyo, Japan).

## 3. Results

There was no significant difference in patients' backgrounds, including mean age, sex, underlying disease, and periampullary diverticulum between the EST and non-EST groups (NS: not significant; Table 1).

The extent of incisions in the EST group was small in 11 patients, medium in 20 patients, and large in one patient. As for other endoscopic procedures, there were no differences in pancreatography, biliary biopsy, bile cytology, pancreatic juice cytology, intraductal ultrasonography of the bile duct, and pancreatic stenting between the EST and non-EST groups. A difference in mean procedure time between the two groups was also not observed (NS; Table 2).

A difference in the type and diameter of stent used was not noted (NS). A stent length of 6 cm was significantly selected more frequently for the non-EST group (*P* = 0.016), whereas a length of 8 cm was more frequently chosen for the EST group (*P* = 0.030; Table 3).

The success rates of drainage for the EST and non-EST groups were 100% (32/32) and 97.2% (35/36), respectively, and were statistically not significant (*P* = 0.95).

**Figure 2:**
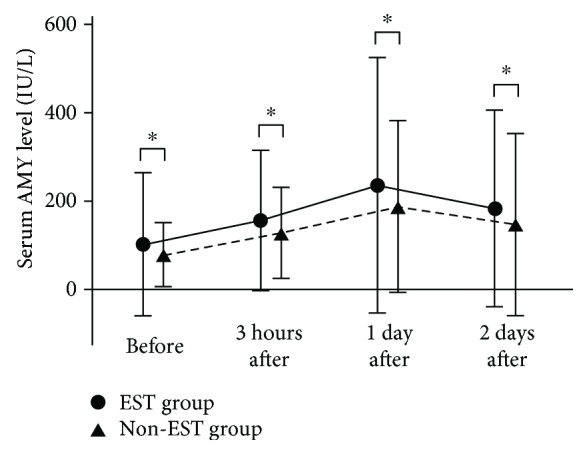
Comparison of mean serum amylase levels between the EST and non-EST groups. ^∗^Not significant.

The incidence of pancreatitis in the EST and non-EST groups was 3.1% (1/32) and 0% (0/36), respectively, and lacked any statistically significant difference (*P* = 0.95). The incidence of hyperamylasemia in the EST and non-EST groups was 12.5% (4/32) and 13.9% (5/36), respectively, and was not significantly different (*P* = 0.85). Serum amylase levels (mean ± SD) before FCSEMS placement, 3 hours after the procedure, the next day, and two days later were 99.9 ± 159.6, 153.7 ± 155.9, 231.5 ± 285.9, and 179.6 ± 143.9 for the EST group and 77.4 ± 71.3, 125.6 ± 101.6, 185.1 ± 192.3, and 145.3 ± 204.1 for the non-EST group, showing no significant differences (NS; Figure 2). The incidence of all AEs in the EST and non-EST groups was 15.6% (5/32) and 13.9% (5/36), respectively, showing a lack of a significant difference (*P* = 0.89). AEs included pancreatitis (*n* = 1), cholecystitis (*n* = 2), and stent migration (*n* = 2) in the EST group and cholecystitis (*n* = 3) and stent migration (*n* = 2) in the non-EST group (Table 4). The severity of pancreatitis, which a patient in the EST group developed, was mild; the FCSEMS used in this patient is a WallFlex Biliary RX stent of 10 mm in diameter and 8 cm in length. Bleeding, perforation, or procedure-related death did not occur in either group.

## 4. Discussion

AEs associated with SEMS placement have included pancreatitis, cholecystitis, and stent migration [15, 24–26], of which pancreatitis may potentially be life-threatening. Previous reports comparing the incidence of pancreatitis in the presence and absence of EST before SEMS placement are shown in Table 5. Two RCTs concluded that EST did not contribute to the prevention of the development of pancreatitis [18, 19]. However, the type of stent used in these studies was PCSEMS with a braded type and a study using FCSEMS has not been reported. FCSEMS is theoretically associated with a higher risk of causing pancreatitis due to occlusion of the pancreatic duct orifice as compared to PCSEMS because it is covered along its entire length. However, we found that the incidence of pancreatitis was not significantly different in the presence and absence of EST, suggesting that EST before FCSEMS for the purpose of prevention of pancreatitis is not necessary. In addition, the incidence of hyperamylasemia and changes in serum amylase levels did not differ between the EST and non-EST groups, suggesting a lack of association between FCSEMS placement and the obstruction of the outflow of pancreatic juice.

However, a pancreatic head tumor was associated with dilatation of the MPD in our study patients, suggesting poor pancreatic juice outflow. Kawakubo et al. [16] investigated risk factors for pancreatitis after SEMS placement using multivariate analysis and identified diseases other than pancreatic cancer and a stent with a high axial force as risk factors, though a procedure without EST was not recognized. Shimizu et al. [17] also used multivariate analysis to investigate risk factors for pancreatitis after SEMS placement and identified diseases other than pancreatic cancer and pancreatography but not a lack of EST as a risk factor. The reason why diseases other than pancreatic cancer were identified as risk factors for pancreatitis was inferred from reports that diseases other than pancreatic cancer were associated with preserved pancreatic exocrine function due to the pancreatic duct not being completely obstructed. On the other hand, pancreatic head cancer often causes atrophy of distal pancreatic parenchyma and declining of pancreatic exocrine function, which may contribute to be less likely to the development post-ERCP pancreatitis. In this study, 64 of 68 patients had pancreatic cancer, which may be one of the reasons for the low incidence of post-ERCP pancreatitis. Therefore, further investigation is required to determine whether EST before FCSEMS placement should be performed or not in diseases other than pancreatic cancer, such as a pancreatic head tumor without MPD dilatation, and bile duct cancer.

Another reason for the low incidence of pancreatitis in this study may be that the axial force of the stents used in this study was relatively low. The concept of the axial force of the stent was proposed by Isayama et al. and defined as the recovery or straightening force when the stent bended [27]. They reported that high axial force was related with the cause of pancreatitis, cholecystitis, and bile duct kinking due to the compression of the pancreatic duct orifice, cystic duct orifice, and bile duct [28]. Furthermore, as described above, Kawakubo et al. examined the risk factors of pancreatitis after SEMS placement using multivariate analysis and identified the stent with a high axial force as a risk factor [16]. Therefore, an ideal stent for the prevention of adverse events including pancreatitis associated with SEMS placement for distal malignant biliary strictures may be a stent with low axial force. It would be desirable to evaluate whether EST is needed for the prevention of pancreatitis when using the stent with a high axial force in the future.

In our present study, a difference in the incidence of other AEs, including bleeding, perforation, stent migration, and cholecystitis, between the EST and non-EST groups was not observed. Bleeding or perforation was not observed in either group. Artifon et al. [18] reported that bleeding or perforation was not observed and the rate of migration was low in 2.7% (1/37) of the non-EST group in an RCT comparing AEs in the presence and absence of EST before PCSEMS placement. However, in the EST group, rates were high for bleeding at 13.5% (5/37), perforation at 10.8% (4/37), and migration at 16.3% (6/37). On the other hand, in a similar RCT comparing AEs in the presence and absence of EST before PCSEMS placement by Hayashi et al. [19], the incidence of bleeding and perforation was 0% (0/100) and 1% (1/100), respectively, for the non-EST group, and 1% (1/100) and 0% (0/100), respectively, for the EST group, showing extremely low incidences in both groups. The reasons for the differences may be ascribed to variations in the incision length of each EST in a setting of high-frequency devices. Whereas the setting of a high-frequency device was in the form of a blended current in our study and that by Hayashi et al. [19], a pure cut current was used in the investigation by Artifon et al. [18]. If EST is performed using a blended current with a small or medium length incision, the risk of bleeding and perforation may be lower. As for stent migration, Nakai et al. [26] showed that EST is not a risk factor; however, CSEMS with a low radial force, chemotherapy, and duodenal invasion are risk factors. EST may be performed in order to allow the easier insertion of instruments when performing a biliary biopsy or brushing cytology before CSEMS placement; performing EST, as necessary, is considered acceptable.

The following limitations were included in our present study. This was a retrospective study in a single institution. The decision to perform EST was made by the attending endoscopist in each case. Various types of FCSEMS were used, and drugs used for all patients for the prevention of pancreatitis were nonuniform and varied. Although there was significant difference in the stent length between the EST group and the non-EST group, it was impossible to identify the reason because of the property of the retrospective study. Additionally, our present results need to be verified by a multicenter RCT in the future. Further validation is also required for the necessity of EST before FCSEMS in cases without MPD dilatation or in those with diseases other than pancreatic cancer.

In summary, our present results suggested that the necessity of EST to prevent pancreatitis before FCSEMS placement in patients having a distal malignant biliary stricture due to pancreatic head tumor was deemed low. Our study also demonstrated that the rate of AEs, including bleeding, perforation, and migration, was not significantly increased by the addition of EST. Therefore, performing EST, when thought necessary, may be acceptable.

## Figures and Tables

**Table 1:** Comparison of patient characteristics between the EST and non-EST groups.

	EST group (*n* = 32)	Non-EST group (*n* = 36)	*P* value
Age (mean ± SD)	71.8 ± 11.3	75.1 ± 11.6	0.240
Sex (male/female)	15/17	22/14	0.239
Pancreatic cancer	30	34	0.693
Metastatic pancreatic cancer	2	2	0.693
Periampullary diverticulum	5	3	0.579

EST: endoscopic sphincterotomy; SD: standard deviation.

**Table 2:** Comparison of endoscopic procedures between the EST and non-EST groups.

	EST group (*n* = 32)	Non-EST group (*n* = 36)	*P* value
Incision range of EST			
Small/medium/large	11/20/1	—	
Pancreatography	14	14	0.684
Biliary biopsy	7	5	0.587
Bile cytology	13	8	0.101
Pancreatic juice cytology	1	1	0.526
IDUS of bile duct	0	1	0.953
Pancreatic stenting	1	0	0.953
Procedure time (min, mean ± SD)	31.1 ± 13.9	29.1 ± 9.8	0.501

EST: endoscopic sphincterotomy; IDUS: intraductal ultrasonography; SD: standard deviation.

**Table 3:** Comparison of placed fully covered self-expandable metal stents between EST and non-EST groups.

	EST group (*n* = 32)	Non-EST group (*n* = 35)	*P* value
WallFlex Biliary RX stent	22	29	0.400
Niti-S COMVI stent	4	4	
X-Suit NIR Biliary Metal stent	2	1	
Hanarostent	1	2	
ZEO stent	1	0	
Bonastent	1	0	
Niti-S SUPREMO-12 stent	1	0	
Stent length			
6 cm	17	29	0.016
7 cm	1	0	
8 cm	14	7	0.030
Stent diameter			
10 mm	31	36	
12 mm	1	0	

EST: endoscopic sphincterotomy.

**Table 4:** Comparison of adverse events between the EST and non-EST groups.

	EST group (*n* = 32)	Non-EST group (*n* = 36)	*P* value
Adverse events (*n* (%))	5 (15.6)	5 (13.9)	0.888
Pancreatitis	1 (3.1)	0 (0)	0.953
Cholecystitis	2 (6.3)	3 (8.3)	0.891
Migration	2 (6.3)	2 (5.6)	0.693
Bleeding	0 (0)	0 (0)	—
Perforation	0 (0)	0 (0)	—

EST: endoscopic sphincterotomy

**Table 5:** Previous reports comparing adverse events in the presence or absence of endoscopic sphincterotomy before biliary metal stent placement.

Author	Year	Study design	Stent type (*n*)	Primary diseases with EST/without EST (*n*)		Adverse events with EST/without EST (%)				
				P-cancer	Others	Pancreatitis	Bleeding	Perforation	Migration	Cholecystitis
Artifon [18]	2008	RCT	PC: 74	30/30	7/7	0/0	13.5/0	10.8/0	16.2/2.7	—
Banerjee [20]	2011	Retro	NC: 70, PC: 34	56	48	3.7/0	18.5/0	—	3.7/3.9	—
Zhou [22]	2012	RCT	NC: 82	11/10	30/31	9.8/31.7	—	—	—	—
Nakahara [21]	2013	Retro	PC: 57, FC: 22	37/36	1/5	2.6/2.4	2.6/0	0/0	2.6/7.3	5.3/4.9
Hayashi [19]	2015	RCT	PC: 200	100/100	0/0	9.4/8.2	1.0/0	0/1.0	—	—
Present study	—	Retro	FC: 68	30/34	2/2	3.1/0	0/0	0/0	6.3/5.6	6.3/5.6

EST: endoscopic sphincterotomy; P-cancer: pancreatic cancer; RCT: prospective randomized controlled trial; Retro: retrospective study; PC: partially covered; NC: noncovered; FC: fully covered.

## Data Availability

The data used to support the findings of this study are available from the corresponding author upon request.

## References

[1] Isayama H., Komatsu Y., Tsujino T. (2002). Polyurethane-covered metal stent for management of distal malignant biliary obstruction. *Gastrointestinal Endoscopy*.

[2] Isayama H., Komatsu Y., Tsujino T. (2004). A prospective randomised study of “covered” versus “uncovered” diamond stents for the management of distal malignant biliary obstruction. *Gut*.

[3] Kubota Y., Mukai H., Nakaizumi A. (2005). Covered Wallstent for palliation of malignant common bile duct stricture: prospective multicenter evaluation. *Digestive Endoscopy*.

[4] Kahaleh M., Tokar J., Conaway M. R. (2005). Efficacy and complications of covered Wallstents in malignant distal biliary obstruction. *Gastrointestinal Endoscopy*.

[5] Nakai Y., Isayama H., Komatsu Y. (2005). Efficacy and safety of the covered WALLSTENT in patients with distal malignant biliary obstruction. *Gastrointestinal Endoscopy*.

[6] Shin H. P., Kim M. H., Jung S. W. (2006). Endoscopic removal of biliary self-expandable metallic stents: a prospective study. *Endoscopy*.

[7] Kasher J. A., Corasanti J. G., Tarnasky P. R., McHenry L., Fogel E., Cunningham J. (2011). A multicenter analysis of safety and outcome of removal of a fully covered self-expandable metal stent during ERCP. *Gastrointestinal Endoscopy*.

[8] Kida M., Miyazawa S., Iwai T. (2011). Endoscopic management of malignant biliary obstruction by means of covered metallic stents: primary stent placement vs. re-intervention. *Endoscopy*.

[9] Ishii K., Itoi T., Sofuni A. (2011). Endoscopic removal and trimming of distal self-expandable metallic biliary stents. *World Journal of Gastroenterology*.

[10] Kogure H., Ryozawa S., Maetani I. (2018). A prospective multicenter study of a fully covered metal stent in patients with distal malignant biliary obstruction: WATCH-2 study. *Digestive Diseases and Sciences*.

[11] Tarnasky P. R., Cunningham J. T., Hawes R. H. (1997). Transpapillary stenting of proximal biliary strictures: does biliary sphincterotomy reduce the risk of postprocedure pancreatitis?. *Gastrointestinal Endoscopy*.

[12] Simmons D. T., Petersen B. T., Gostout C. J., Levy M. J., Topazian M. D., Baron T. H. (2008). Risk of pancreatitis following endoscopically placed large-bore plastic biliary stents with and without biliary sphincterotomy for management of postoperative bile leaks. *Surgical Endoscopy*.

[13] Jeong Y. W., Shin K. D., Kim S. H. (2009). The safety assessment of percutaneous transhepatic transpapillary stent insertion in malignant obstructive jaundice: regarding the risk of pancreatitis and the effect of preliminary endoscopic sphincterotomy. *Korean Journal of Gastroenterology*.

[14] Cui P. J., Yao J., Zhao Y. J., Han H. Z., Yang J. (2014). Biliary stenting with or without sphincterotomy for malignant biliary obstruction: a meta-analysis. *World Journal of Gastroenterology*.

[15] Cote G. A., Kumar N., Ansstas M. (2010). Risk of post-ERCP pancreatitis with placement of self-expandable metallic stents. *Gastrointestinal Endoscopy*.

[16] Kawakubo K., Isayama H., Nakai Y. (2012). Risk factors for pancreatitis following transpapillary self-expandable metal stent placement. *Surgical Endoscopy*.

[17] Shimizu S., Naitoh I., Nakazawa T. (2013). Predictive factors for pancreatitis and cholecystitis in endoscopic covered metal stenting for distal malignant biliary obstruction. *Journal of Gastroenterology and Hepatology*.

[18] Artifon E. L. A., Sakai P., Ishioka S. (2008). Endoscopic sphincterotomy before deployment of covered metal stent is associated with greater complication rate: a prospective randomized control trial. *Journal of Clinical Gastroenterology*.

[19] Hayashi T., Kawakami H., Osanai M. (2015). No benefit of endoscopic sphincterotomy before biliary placement of self-expandable metal stents for unresectable pancreatic cancer. *Clinical Gastroenterology and Hepatology*.

[20] Banerjee N., Hilden K., Baron T. H., Adler D. G. (2011). Endoscopic biliary sphincterotomy is not required for transpapillary SEMS placement for biliary obstruction. *Digestive Diseases and Sciences*.

[21] Nakahara K., Okuse C., Suetani K. (2013). Covered metal stenting for malignant lower biliary stricture with pancreatic duct obstruction: is endoscopic sphincterotomy needed?. *Gastroenterology Research and Practice*.

[22] Zhou H., Li L., Zhu F., Luo S. Z., Cai X. B., Wan X. J. (2012). Endoscopic sphincterotomy associated cholangitis in patients receiving proximal biliary self-expanding metal stents. *Hepatobiliary & Pancreatic Diseases International*.

[23] Cotton P. B., Lehman G., Vennes J. (1991). Endoscopic sphincterotomy complications and their management – an attempt at consensus. *Gastrointestinal Endoscopy*.

[24] Isayama H., Kawabe T., Nakai Y. (2006). Cholecystitis after metallic stent placement in patients with malignant distal biliary obstruction. *Clinical Gastroenterology and Hepatology*.

[25] Fumex F., Coumaros D., Napoleon B. (2006). Similar performance but higher cholecystitis rate with covered biliary stents: results from a prospective multicenter evaluation. *Endoscopy*.

[26] Nakai Y., Isayama H., Kogure H. (2014). Risk factors for covered metallic stent migration in patients with distal malignant biliary obstruction due to pancreatic cancer. *Journal of Gastroenterology and Hepatology*.

[27] Isayama H., Nakai Y., Toyokawa Y. (2009). Measurement of radial and axial forces of biliary self-expandable metallic stents. *Gastrointestinal Endoscopy*.

[28] Isayama H., Nakai Y., Hamada T., Matsubara S., Kogure H., Koike K. (2016). Understanding the mechanical forces of self-expandable metal stents in the biliary ducts. *Current Gastroenterology Reports*.

